# Association between polymorphisms in ERCC2 gene and oral cancer risk: evidence from a meta-analysis

**DOI:** 10.1186/1471-2407-13-594

**Published:** 2013-12-12

**Authors:** Enjiao Zhang, Zhigang Cui, Zhongfei Xu, Weiyi Duan, Shaohui Huang, Xuexin Tan, Zhihua Yin, Changfu Sun, Li Lu

**Affiliations:** 1 Department of Oral and Maxillofacial Surgery, School of Stomatology, China Medical University, Nanjing North Street, Shenyang, Heping District 110002, People’s Republic of China; 2 China Medical University, Shenyang, PR 110001, China; 3 Department of Epidemiology, School of Public Health, China Medical University, Shenyang, PR 110001, China

## Abstract

**Background:**

*Excision repair cross-complementing group 2* (*ERCC2*) plays important roles in the repair of DNA damage and adducts. Single nucleotide polymorphisms (SNPs) of *ERCC2* gene are suspected to influence the risks of oral cancer. We performed a meta-analysis to systematically summarize the possible association of *ERCC2* rs1799793 and rs13181 polymorphisms with oral cancer risks.

**Methods:**

We retrieved the relevant articles from PubMed and Embase databases. Studies were selected using specific criteria. ORs and 95% CIs were calculated to assess the association. All analyses were performed using the Stata software.

**Results:**

Six studies were included in this meta-analysis. There were no significant associations between *ERCC2* rs1799793 and rs13181 polymorphism with overall oral cancer risk. In the stratified analysis by ethnicity, no significant associations were found. In the stratified analysis by tumor type, the risk of oral leukoplakia was significant associated with rs13181 polymorphism (AC vs. AA: OR = 1.28, 95% CI = 1.01-1.62, P = 0.546 for heterogeneity, I^2^ = 0.0%; CC vs. AA: OR = 1.94, 95% CI = 0.99-3.79, P = 0.057 for heterogeneity, I^2^ = 60.1%; dominant model AC + CC vs. AA: OR = 1.35, 95% CI = 1.08–1.69, P = 0.303 for heterogeneity, I^2^ = 17.6%; allele C vs. A: OR = 1.38, 95% CI = 1.04–1.82. P = 0.043 for heterogeneity, I^2^ = 56.4%).

**Conclusion:**

Rs13181 in *ERCC2* gene might be associated with oral leukoplakia risk.

## Background

An estimated 263,900 new cases and 128,000 deaths from oral cavity cancer (including lip cancer) occurred in 2008 worldwide [1]. Its increasing incidence and mortality rates during the last two decades pose a big challenge to scientists and doctors. A review highlighted the strength of the association of several of the risk factors (e.g., tobacco and alcohol use, and diet) related to oral and pharyngeal cancers [2]. Early premalignant oral lesions, such as leukoplakia, appear as a white patch in the oral cavity of chewing and tobacco smoking, and five to ten percent of them progress to malignancy [3]. Therefore, the identification of biomarkers for screening the high-risk individuals for increased predisposition to cancer is very important for prevention of cancer.

Environmental carcinogens contained in air pollution or tobacco smoking fumes, which are suggested to be important risk factors for oral cancer, could cause many types of DNA damages such as forming DNA adducts, cross-links and unrepaired DNA damage can result in cell apoptosis or unregulated cell growth and may eventually lead to cancer. The various DNA repair pathways play important roles in the genomic stability, thus defending against carcinogenesis. Individuals with suboptimal DNA repair capacity are at increased risk of smoking-related cancers, such as lung cancer and squamous cell carcinoma of the head and neck [4,5]. There has been increasing evidence that DNA damage plays a critical role in the carcinogenesis of most cancers and DNA repair genes are considered key genes associated with the onset of cancer [6-8]. There are at least four pathways of DNA repair on specific types of DNA damage [9]. Tobacco-induced DNA adducts are primarily removed by nucleotide excision repair (NER) pathway. The variation in DNA repair capacity may due to the single nucleotide polymorphisms (SNP) in DNA repair genes. So it is of utmost importance to investigate the SNPs in genes involved in NER pathway to understand the etiology of oral cancer.

*Excision repair cross-complimentary group 2* (*ERCC2*) is an important DNA repair gene in NER pathway. *ERCC2* is located in chromosome 19q13.2-13.3 and codes for an evolutionarily conserved helicase, a subunit of TFIIH complex, which is essential for NER. SNPs in exons of DNA repair genes may influence their protein activity, resulting in differences of individual NER and DNA repair capacity that may affect the susceptibility of diseases. The common polymorphisms in exons of *ERCC2* gene is at codon 751 (A > C substitution at nucleotide position 35931, exon 23, Lys > Gln, rs13181) and codon 312 (G > A substitution at position 23951, exon 10, Asp > Asn, rs1799793). To date, there are studies reporting the association between polymorphisms of *ERCC2* codon 312 and 751with oral cancer risk but these published data were contradictory [10-15]. Until now, there was no meta-analysis or systematic review on the risk of oral cancer with *ERCC2* polymorphism. So we perform an updated meta-analysis on all available case–control studies to assess the oral cancer risk with rs13181 and rs1799793 in *ERCC2* gene.

## Methods

### Data sources

We retrieved the articles using the following terms “Excision repair cross-complimentary group 2 or *ERCC2* or Xeroderma pigmentosum D or XPD” and “oral cancer or oral carcinoma” from PubMed and Embase (Last search was updated on May 2013). We evaluated potentially relevant publications by examining their titles and abstracts and all studies matching the eligible criteria were retrieved.

### Study selection and data extraction

Eligible studies were selected according to the following explicit inclusion criteria: (a) evaluation of the rs13181 and/or rs1799793 polymorphism and oral cancer or oral leukoplakia risks, (b) using the methodology of a case–control study. (c) There was sufficient published data for the computation of odds ratios (ORs) with 95% confidence intervals (95% CIs), for example there are number of cases and controls with different genotypes or alleles in published paper to calculate ORs and their 95% CIs.

Duplicate and obviously unrelated articles were eliminated by a single author (E.Z.). Abstracts of the remaining articles were examined independently by two authors (E.Z. and Z.C.) to determine whether the full-text article should be sought. The following information was obtained from each publication: first author’s name, publication year, country origin, ethnicity, case characteristics, total number of cases and controls, and numbers of each group with rs13181 and rs1799793 genotypes, respectively.

### Statistical methods

We first assessed Hardy-Weinberg equilibrium using Chi-square test in control groups for each included study. ORs and their 95% CIs were calculated to evaluate the association between *ERCC2* SNPs and cancer risks. Pooled ORs were calculated from combination of each study by heterozygote comparison (GA vs. GG for rs1799793; AC vs. AA for rs13181), homozygote comparison (AA vs. GG for rs1799793; CC vs. AA for rs13181), dominant model (GA + AA vs. GG for rs1799793; AC + CC vs. AA for rs13181), recessive model (AA vs. GA + GG for rs1799793; CC vs. AC + AA for rsrs13181) and allelic model (A vs. G for rs1799793; C vs. A for rs13181) respectively. For each genetic comparison model, subgroup analysis according to ethnicity was investigated to estimate ethnic-specific ORs for Asian population, but not for Caucasian population because there was only one paper in Caucasians. Meanwhile stratified analyses by tumor type were also applied for each genetic comparison model. Values of Akaike’s Information Criterion (AIC) are reported for model comparison, with the best models showing the smallest AIC [16].

We assessed the between-study heterogeneity by Cochran’s Q test and quantified by I^2^ (a significance level of P < 0.10 and/or I^2^ ≥ 50%). If the P value is >0.05 of the Q test, the summary OR estimate of each study was calculated by the fixed-effect model. Otherwise, the random-effect model was used. The effect of publication bias was examined by inverted funnel plots and the Egger’s test. The significance of the intercept was determined by the t test as suggested by Egger’s test. All of P values were two-sided and all analyses were performed using the Stata software version 11.0 (Stata Corp, College station, TX).

## Results

### Characteristics of included studies

**Figure 1:**
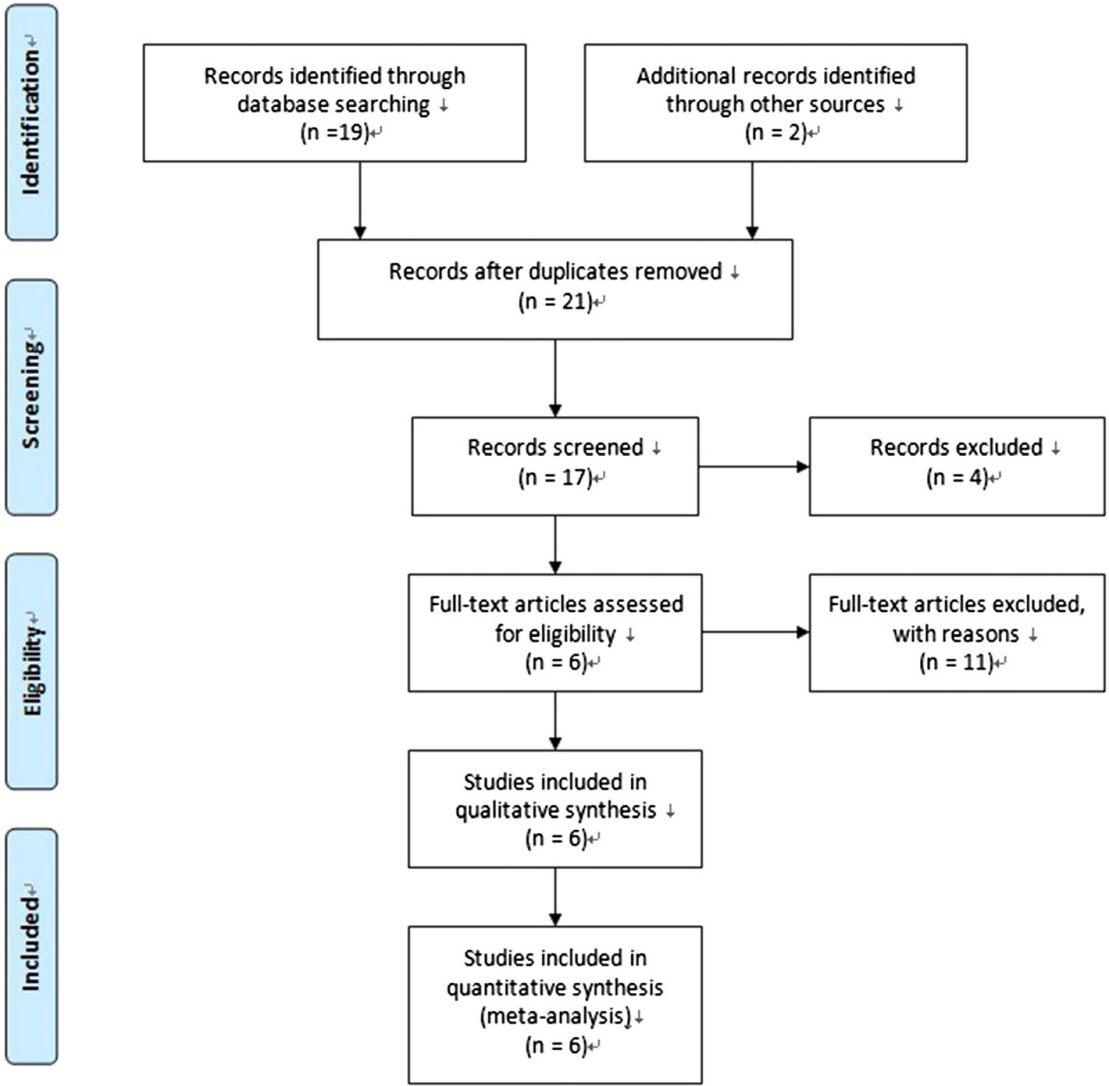
Flow chart of the study selection process.

According to these criteria, a total of 17 articles were eligible. One study of review, two studies on cancer prognosis and three studies about cell line were excluded. Five studies were excluded because of no cancer risk and data missing. Finally 6 articles were included and used in quantitative synthesis for systematic review [10-15]. Flow chart of the study selection process was shown in Figure 1.

The characteristics of selected studies are summarized in Table 1. There were one study of European and five studies of Asians. There are three studies about *ERCC2* rs1799793 SNP, including 742 cases and 738 controls. There were 1202 cases and 1145 controls in 6 studies for *ERCC2* rs13181 SNP. Among three studies of *ERCC2* rs1799793 polymorphisms, one study included the association between this polymorphism with oral cancer risk and all of the three studies contain the association between the polymorphism and oral leukoplakia risk. For rs13181 polymorphism, data sets about the risk of oral cancer and oral leukoplakia were both four. The polymerase chain reaction–restriction fragment length polymorphism (PCR-RFLP) method was the most common technique used for analyzing the genotype frequencies of the two SNPs. The distributions of genotypes in the controls were all in Hardy-Weinberg equilibrium (HWE).

**Table 1:** Characteristics of all studies in meta-analysis

**Author, year**	**Country**	**Ethnicity**	**Age (case/control)**	**Case gender**	**Control gender**	**SNP**	**Case type**	**No. (case/control)**	**Case**			**Control**			
									**GG/AA**	**GA/AC**	**AA/CC**	**GG/AA**	**GA/AC**	**AA/CC**	**HWE(P)**
				**(Male/female)**	**(Male/female)**										
Mahimkar MB (2010) [10]	India	Asian	39.0 ± 13.0/39.0 ± 13.0	60/6	60/2	Rs1799793	OL	66/62	23	13	4	23	21	1	0.13
						Rs13181	OL	66/62	26	20	10	29	23	2	0.32
Wang Y (2007) [11]	America	Caucasian	58.3 ± 12.8/59.7 ± 11.0	81/63	162/126	Rs1799793	OL	144/288	50	59	16	140	109	29	0.26
						Rs13181	OL	144/288	46	77	21	120	132	28	0.34
Majumder M (2007) [12]	India	Asian	49.0 ± 11.9/47.0 ± 10.3	196/28	302/87	Rs1799793	OL	224/388	117	89	18	205	146	36	0.18
							OSCC	308/388	152	119	34	205	146	36	0.18
							Total	532/388	269	208	52	205	146	36	0.18
						Rs13181	OL	224/388	105	98	21	190	158	40	0.40
							OSCC	308/388	158	125	26	190	158	40	0.40
							Total	532/388	263	223	47	190	158	40	0.40
Kietthubthew S (2006) [13]	Thailand	Asian	67.1/68.4	77/29	91/73	Rs13181	OSCC	112/192	83	21	1	126	36	2	0.75
Bau DT (2007) [14]	China	Asian	53.0 ± 10.1/ 54.4 ± 12.1	None	None	Rs13181	OC	154/105	134	18	2	89	15	1	0.68
Ramachandran S (2006) [15]	India	Asian	None	None	None	Rs13181	OC	110/110	49	46	15	71	31	8	0.09
							OL	84/110	41	29	14	71	31	8	0.09
							Total	194/110	90	75	29	71	31	8	0.09

OL: Oral leukoplakia, OSCC: Oral squamous cell cancer, OC: oral cancer.

Total is the sum of different case type about the same SNP in each study.

### ERCC2 rs1799793 SNP

The A allele frequency of the *ERCC2* rs1799793 polymorphism among the controls across different ethnicities ranged from 0.26 to 0.30. The average A allele frequencies in Asian and Caucasians populations were 27.0 and 30.0%, respectively. Heterogeneity between studies was not observed so the fixed-effect model was conducted. The overall ORs with its 95% CIs didn’t show statistically association between rs1799793 polymorphism and oral cancer risk (GA vs. GG: OR = 1.14, 95% CI = 0.91-1.43, P = 0.182 for heterogeneity, I^2^ = 41.2%; AA vs. GG: OR = 1.27, 95% CI = 0.87-1.86, P = 0.436 for heterogeneity, I^2^ = 0%; dominant model GA + AA vs. GG: OR = 1.16, 95% CI = 0.94-1.44, P = 0.268 for heterogeneity, I^2^ = 24.0%; recessive model AA vs. GA + GG: OR = 1.18, 95% CI = 0.82-1.70, P = 0.406 for heterogeneity, I^2^ = 0%; allele A vs. G: OR = 1.13, 95% CI = 0.96–1.34, P = 0.491 for heterogeneity, I^2^ = 0%) (Table 2). Because there was only one study among Caucasian population and one study on oral squamous cell cancer, the stratified analyses were not conducted in rs1799793 polymorphism.

**Table 2:** **Association between *ERCC2 *** **polymorphisms with oral cancer risks**

	**No of studies**	**Fixed-effect**	**Random-effect**	**Phet**	**I-squared (%)**
Rs1799793					
GA vs. GG	3	1.14[0.91,1.43]	1.13[0.80,1.61]	0.182	41.2
AA vs. GG	3	1.27[0.87,1.86]	1.24[0.87,1.85]	0.436	0.0
GA + AA vs. GG	3	1.16[0.94,1.44]	1.17[0.89,1.55]	0.268	24.0
AA vs. GA + GG	3	1.18[0.82,1.70]	1.16[0.81,1.68]	0.406	0.0
A vs. G	3	1.13[0.96,1.34]	1.13[0.96,1.34]	0.491	0.0
Rs13181					
AC vs. AA	6	1.16[0.96,1.40]	1.17[0.90,1.51]	0.171	35.5
CC vs. AA	6	1.42[1.03,1.96]	1.71[0.92,3.20]	0.044	56.1
AC + CC vs. AA	6	1.19[1.00,1.43]	1.24[0.92,1.67]	0.045	55.8
CC vs. AC + AA	6	1.29[0.95,1.76]	1.48[0.87,2.52]	0.101	45.7
C vs. A	6	1.17[1.02,1.34]	1.23[0.94,1.62]	0.011	66.1

Phet: P value for heterogeneity test.

No publication bias was detected by either the inverted funnel plot or Egger’s test. The shapes of the funnel plot for the comparison of the G allelic and the A allelic of rs1799793 SNP seemed approximately symmetrical and P value of the Egger’ test was not statistical significant (t = 0.08, P = 0.940).

### ERCC2 rs13181 SNP

The C allele frequency of *ERCC2* rs13181 polymorphism among the controls across different ethnicities ranged from 0.08 to 0.34. The average C allele frequencies in Asian and Caucasians populations were 19.4% and 34.0%, respectively. There was almost no significant heterogeneity in the analyses. The associations between rs13181 polymorphism and overall oral cancer risk were not statistically significant (AC vs. AA: OR = 1.16, 95% CI = 0.96-1.40, P = 0.171 for heterogeneity, I^2^ = 35.5%; CC vs. AA: OR = 1.71, 95% CI = 0.92-3.20, P = 0.044 for heterogeneity, I^2^ = 56.1%; dominant model AC + CC vs. AA: OR = 1.24, 95%CI = 0.92–1.67, P = 0.045 for heterogeneity, I^2^ = 55.8%; recessive model CC vs. AC + AA: OR = 1.29, 95% CI = 0.95–1.76, P = 0.101 for heterogeneity, I^2^ = 45.7%; allele C vs. A: OR = 1.23, 95% CI = 0.94–1.62, P = 0.011 for heterogeneity, I^2^ = 66.1%). The AIC values of heterozygote model, variant homozygote model, dominant model, recessive model and allelic model were 88.4, 104.6, 109.2, 87.2 and 118.0, showing that the recessive model may be better than other models.

**Figure 2:**
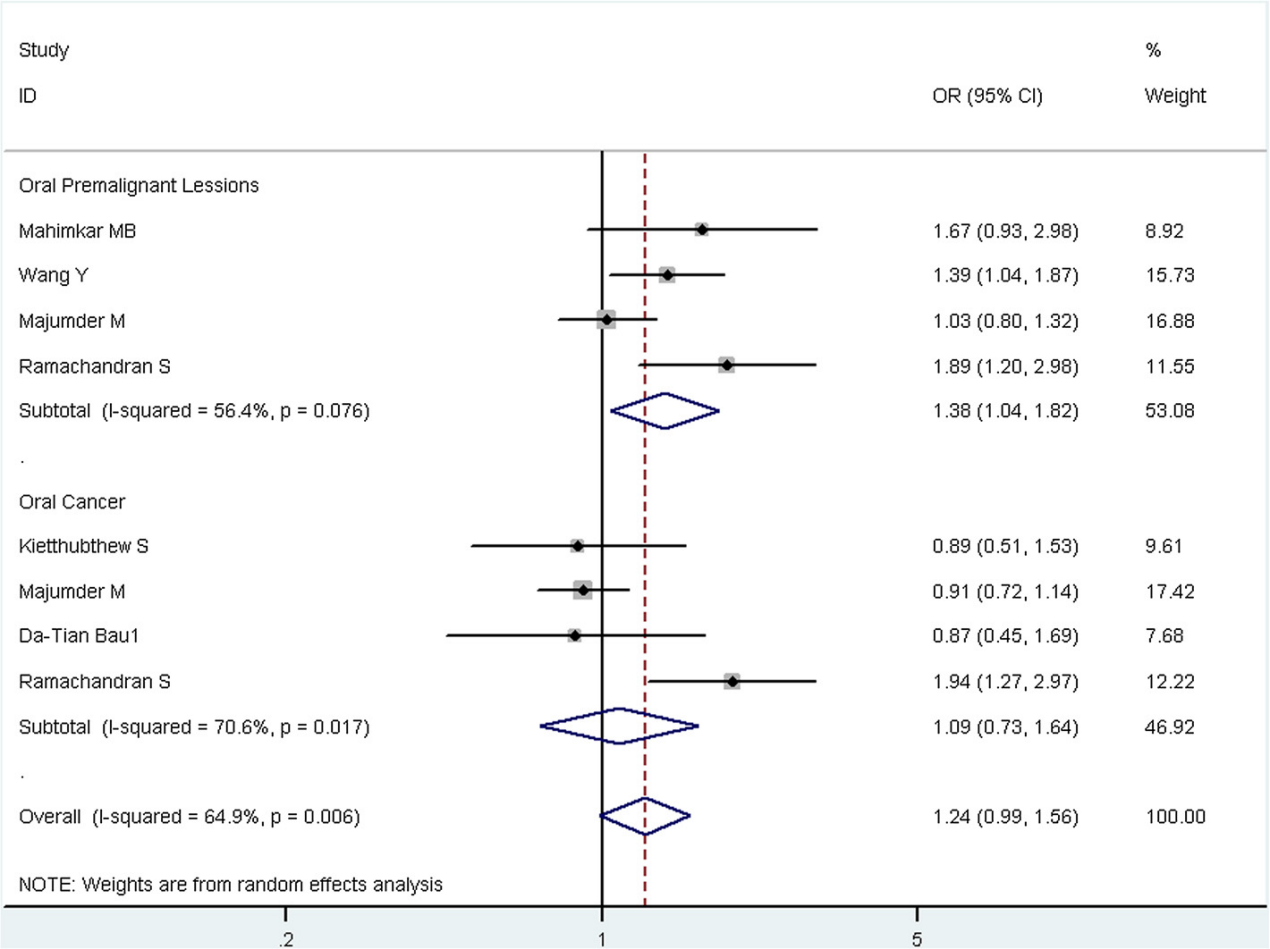
**Meta-analysis of the association between *ERCC2 *** **rs13181 polymorphism and oral cancer risk stratified by case type under the allele model (C versus A).**

**Figure 3:**
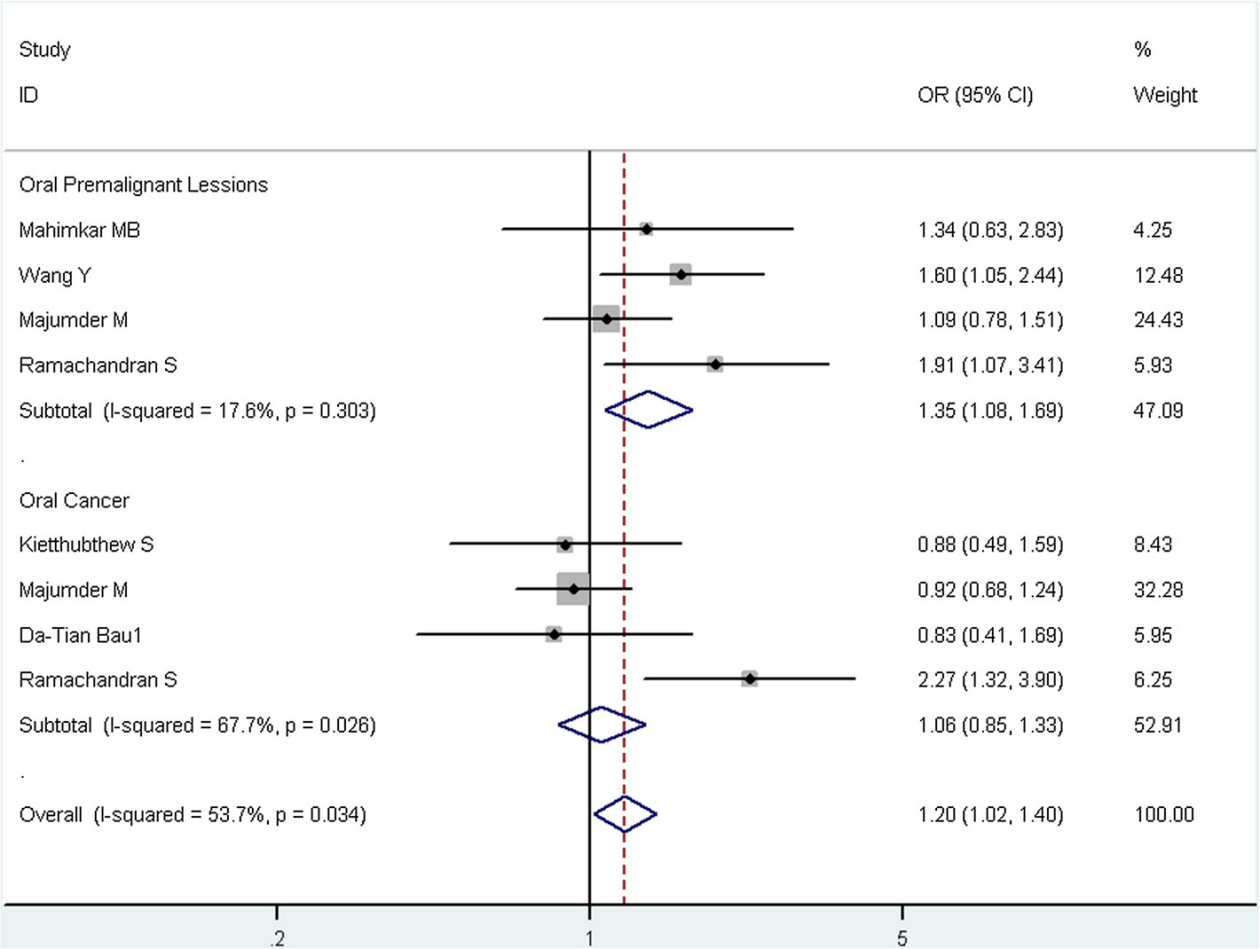
**Meta-analysis of the association between *ERCC2 *** **rs13181 polymorphism and oral cancer risk stratified by case type under the dominant model (AC + CC versus AA).**

Stratified analyses were conducted for rs13181 polymorphism by ethnicity and tumor type (Table 3). In the stratified analysis by ethnicity, no significant associations were found among Asians. However, the subgroup analysis in Caucasians was not further performed because there was only one study from Caucasians. In the stratified analysis by tumor type, the risk of oral leukoplakia was significant associated with rs13181 polymorphism (AC vs. AA: OR = 1.28, 95% CI = 1.01-1.62, P = 0.546 for heterogeneity, I^2^ = 0.0%; CC vs. AA: OR = 1.94, 95% CI = 0.99-3.79, P = 0.057 for heterogeneity, I^2^ = 60.1%; dominant model AC + CC vs. AA: OR = 1.35, 95% CI = 1.08–1.69, P = 0.303 for heterogeneity, I^2^ = 17.6%; allele C vs. A: OR = 1.38, 95% CI = 1.04–1.82. P = 0.043 for heterogeneity, I^2^ = 56.4%). There was no evidence for the influence of rs13181 polymorphism on oral cancer susceptibility. Figure 2 showed the meta-analysis results of the association between *ERCC2* rs13181 polymorphism and oral cancer risk stratified by case type under the allele model (C versus A) from random effects analysis. Figure 3 are the results of the association under the dominant model (AC + CC versus AA) from fixed effects analysis.

**Table 3:** **Pooled ORs and 95% CIs for *ERCC2 *** **rs13181 polymorphism of stratified meta-analysis**

**Subgroup**	**Genotype**	**No of studies**	**Test of association**				**Test of heterogeneity**		
			**OR(95% CI)**	**Z**	**P-value**	**Model**	***χ***^**2**^	**P-value**	**I**^**2**^**(%)**
Asian	AC vs. AA	4	1.16[0.88,1.36]	0.83	0.405	F	5.81	0.121	48.3
	CC vs. AA	4	1.33[0.59,3.01]	0.69	0.489	R	1.41	0.098	52.3
AC + CC vs. AA		4	1.13[0.75,1.71]	0.59	0.555	R	8.79	0.032	65.9
CC vs. AC + AA		4	1.08[0.74,1.56]	0.39	0.694	F	4.36	0.225	61.2
	C vs. A	4	1.12[0.76,1.65]	0.56	0.576	R	10.90	0.012	72.5
Oral leukoplakia	AC vs. AA	4	1.28[1.01,1.62]	2.01	0.045	F	2.13	0.546	0.0
	CC vs. AA	4	1.94[0.99,3.79]	1.95	0.052	R	7.52	0.057	60.1
AC + CC vs. AA		4	1.35[1.08,1.69]	2.60	0.009	F	3.64	0.303	17.6
CC vs. AC + AA		4	1.67[0.90,3.13]	1.63	0.102	R	7.20	0.066	58.3
	C vs. A	4	1.38[1.04,1.82]	2.26	0.024	R	6.88	0.043	56.4
Oral cancer	AC vs. AA	4	1.10[0.73,1.65]	0.45	0.656	R	7.02	0.071	57.3
	CC vs. AA	4	1.07[0.69,1.69]	0.31	0.758	F	5.26	0.154	43.0
AC + CC vs. AA		4	1.11[0.71,1.75]	0.46	0.643	R	9.29	0.026	67.7
CC vs. AC + AA		4	1.01[0.66,1.55]	0.06	0.951	F	3.13	0.371	4.3
	C vs. A	4	1.09[0.73,1.64]	0.44	0.661	R	10.22	0.064	55.0

OR, odds ratio; vs, versus; R, random effect model; F, fixed effect model.

No publication bias was indicated according to the results of the inverted funnel plot, Begg’s test and Egger’s test (data not shown).

## Discussion

It is well known that individual susceptibility plays important role in the development of most cancers. Polymorphisms of genes involved in carcinogenesis may have accounted for the susceptibility. Therefore, genetic susceptibility, especially single nucleotide polymorphism (SNP), to cancer has been a research focus in scientific community. Understanding the genetic background and etiology of oral cancer is essential for both the risk assessment and findings of effective methods of prevention and treatment. Recent genetic association studies on oral cancer risks have focused on the effects of single nucleotide polymorphisms in *Excision repair cross-complimenting group 2* (*ERCC2*) gene, namely *Xeroderma pigmentosum D* (*XPD*), is an important DNA repair gene in nucleotide excision repair (NER) pathway which could repair a wide variety of structurally DNA lesions, including bulky adducts, cross-links [17], oxidative DNA damage, thymidine dimers [18] and alkylating damage [19]. SNPs in exons of DNA repair genes may influence their protein activity, resulting in differences of individual NER and DNA repair capacity (DRC) that may affect the susceptibility of oral cancer. The two SNPs analyzed in the present study were the common SNPs in exons of *ERCC2* gene. SNP rs1799793 is G > A substitution at *ERCC2* codon 312 (exon 10, Asp > Asn) and rs13181 is A > C substitution at *ERCC2* codon 751 (exon 23, Lys > Gln). Growing number of studies have been done to examine the relationship between these two SNPs and the risks of oral cancer [10-15]. However, the results are inconclusive. For the associations of *ERCC2* polymorphisms with cancers, the negative findings may result from the low statistical power of available studies now. To better understanding of the association between these polymorphisms and oral cancer risk, a meta-analysis with larger sample and subgroup analysis is necessary. In the present meta-analysis, the statistical power was increased by combining the results of six included studies. The findings from this meta-analysis suggested that there was a significant association between rs13181 polymorphism in *ERCC2* gene and risk of oral cancer, which provided new evidence for the susceptibility and etiology of oral cancer.

The current study is the first meta-analysis of the association between *ERCC2* rs1799793 and rs13181 polymorphisms with the risk of oral cancer. This meta-analysis suggested that rs13181 (*ERCC2* Lys751Gln) might be associated with oral leukoplakia risk. There were studies suggesting that SNP at amino acid 751 of *ERCC2* may play an important role in *ERCC2* protein activity [20]. The *ERCC2* 751 polymorphism (rs13181) was associated with higher levels of chromatic aberrations [21] and DNA adducts levels [22]. It was reported that *ERCC2* 751(rs13181) AC/CC genotypes were significantly defective in NER [23] and had a modulating effect on DRC [24]. These results suggested that *ERCC2* 751 polymorphism (rs13181) could result in a defect in NER and deficient DRC that may be responsible for increased susceptibility of oral cancer.

Despite our efforts in performing a comprehensive analysis, some limitations exist in our meta-analysis. First, our analysis used published international studies, which could arise publication bias, although the results for publication bias in our study were not statistically significant. Second, lack of the original data of available studies limited our further evaluation of potential interactions, such as age, gender, family history, environmental factors and lifestyle. Third, in stratified analysis we only studied the association between *ERCC2* rs13181 polymorphism and oral cancer in Asians but could not evaluate the association in Caucasians because of the limited studies from Caucasian population. Until now, there was only one relevant study found from Caucasians, and a precise estimation on the association in Caucasians is difficult to make. Therefore, more studies are needed to provide more evidence on the association between *ERCC2* polymorphisms and oral cancer risks in Caucasians and other ethnic populations.

In conclusion, our meta-analysis supported that the rs13181 polymorphism in *ERCC2* gene more likely contribute to the increasing risk of oral leukoplakia. Future well-designed and larger population studies, especially in Caucasians and other ethnic populations are of great value to confirm these findings. Moreover, combination of genetic factors together with environmental exposures should also be considered.

## Conclusion

Rs13181 in *ERCC2* gene might be associated with oral leukoplakia risk.

## Competing interests

The authors declare that they have no competing interests.

## Authors’ contributions

EZ participated in extracting the data, performing the statistical analysis and drafting the manuscript. ZC, ZX and WD participated in study selection, data extraction and drafting the manuscript. SH, XT and ZY collected and extracted the data. CS and LL conceived of the study and participated in drafting the manuscript. All authors read and approved the final manuscript.

## Pre-publication history

The pre-publication history for this paper can be accessed here:

http://www.biomedcentral.com/1471-2407/13/594/prepub
